# Assessing tuberculosis stigma: a pooled multi-country descriptive analysis of 17 high-burden countries using a standardised tool

**DOI:** 10.3389/fpubh.2026.1799432

**Published:** 2026-05-07

**Authors:** Wai Man Peter Mok, James Malar, Viorel Soltan, Caoimhe Smyth, Kinz Ul Eman, Aris Subakti, Rhea Lobo, Tanaka Mukuhwa, Donald Tobaiwa, Maxime Lunga, Vladyslav Denysenko, Bertille Kayowa, Deliana Garcia, Choub Sok Chamreun

**Affiliations:** 1 StopTB Partnership, Le Grand-Saconnex, Switzerland; 2 Dopasi Foundation, Islamabad, Pakistan; 3 Penabulu Foundation, Jakarta, Indonesia; 4 TBpeople, Mumbai, India; 5 Zvandiri, Mutare, Zimbabwe; 6 Jointed Hands, Harare, Zimbabwe; 7 Club des Amis Damien, Kinshasa, Democratic Republic of Congo; 8 100% Life, Kyiv, Ukraine; 9 Global TB Caucus, Austin, TX, United States; 10 KHANA, Phnom Penh, Cambodia

**Keywords:** community-led, Global Fund, self stigma, stigma, stigma in health care, stigma measurement, Stop TB Partnership, tuberculosis

## Abstract

Tuberculosis (TB) stigma continues to impede equitable access to prevention, diagnosis, treatment, and care, undermining efforts to end the epidemic. Despite commitments to eliminate TB stigma in the 2023 United Nations Political Declaration on TB, implementation of evidence-based stigma reduction interventions remains constrained by limited systematic and comparable data. This community-led paper describes findings from 17 national TB stigma assessments conducted using validated stigma measurement scales and guided by the World Health Organization social-ecological model to generate cross-country evidence for action. Across the 17 countries—representing 38% of the estimated global TB burden in 2024—a total of 26,040 individuals participated in the quantitative components. Participants included people with TB (56%, *n* = 14,685), family members (18%, *n* = 4,662), community members living in the same areas (16%, *n* = 4,206), and healthcare workers (10%, *n* = 2,487). The most frequently reported contexts where people with TB reported stigma inhibited access to services were observed in the community (25%) and self-stigma (23%). These were followed by stigma in health facilities (18%), homes and families (18%), and workplaces (16%). Common drivers included self-isolation among people with TB, concealment of TB status by families, community avoidance behaviours (e.g., reluctance to share food or drink), and healthcare workers’ endorsement of forced isolation during treatment. Thirty percent (30%) of healthcare workers reported experiencing stigma, mainly from colleagues. Stigma occurred most often before presentation at health facilities—particularly during symptom recognition, disclosure, and treatment initiation and adherence—and disproportionately affected women and underserved populations. Although most countries reported protective laws and policies—generally embedded in broader health or constitutional frameworks rather than TB-specific legislation—implementation and enforcement were frequently inadequate, particularly regarding workplace protections, privacy, non-discrimination, and safeguards against involuntary isolation. These findings confirm that stigma remains a major barrier to TB care and highlight the need to scale up context-responsive stigma reduction strategies. Priorities include targeting critical settings and stages of the TB care continuum, addressing prevalent stigma drivers, strengthening enforcement of legal protections, prioritizing disproportionately affected populations, and promoting meaningful co-creation and leadership by people affected by TB.

## Highlights

- Systematic, nationwide TB stigma assessments provide the essential evidence needed to design and implement targeted, evidence-based interventions across the TB care cascade, diverse settings, and underserved populations.
- Countries with high proportions of missing people with TB disease should partner with people affected by TB to conduct standardized stigma assessments every three to 5 years to monitor progress, evaluate intervention impact, and continuously refine national responses.
- Reducing stigma is fundamental to achieving effective TB prevention, diagnosis, and treatment—including latent TB infection and the introduction of future tools such as vaccines—and to strengthening global health security.
- Communities of people affected by TB and underserved populations, experience disproportionate levels of TB-related stigma, require tailored, context-specific, nuanced population specific approaches to ensure equitable, people-centered care and be supported to lead these initiatives.
- Sustained investment, accountability, and inclusion of stigma indicators in global monitoring frameworks—such as World Health Organization’s (WHO) Global TB Report—together with cross-country collaboration and learning, are critical to institutionalizing stigma reduction and accelerating progress toward ending both TB stigma and TB itself.

## 1 Introduction

Although tuberculosis (TB) has been a curable disease for decades, it continues to impact millions and cause more than one million deaths annually (1). TB remains the world’s leading infectious disease killer. As reported in 2025, global progress remains alarmingly off track: TB incidence declined only 12% by 2024—far from the 50% reduction targeted by 2025 (1).

A major obstacle to achieving global TB targets is the difficulty in finding all people with TB—both those with active disease and those with latent infection—and ensuring they access and complete treatment.^1^ Equally critical is addressing demographic, social and cultural barriers that hinder timely access to TB prevention, diagnosis, treatment, care and support (1). Among these, stigma has emerged as the most consistently reported and deeply entrenched barrier (2–5).

Stigma was first defined by Erving Goffman as an undesirable attribute that an individual possesses, thus reducing that individual’s status in the eyes of society (6). This was further defined in the context of health (7, 8) and later TB (9, 10). Discrimination—the behavioural manifestation of stigma—leads to unfair or unequal treatment of people with TB (PWTB), impeding both service access and provision. TB-related stigma may occur at multiple points along the care continuum: before diagnosis (when recognising symptoms and seeking care), during diagnosis and treatment, and after completion of therapy. It may manifest within families, communities, workplaces, and health facilities, and often includes self-stigma internalised by PWTB. The same dynamics can also discourage people with latent TB infection from seeking preventive treatment.

Stigma has increasingly been recognised as a determinant of health outcomes, including for TB (11). In low- and middle-income countries, stigma is the most common barrier to active case finding, cited by both PWTB and healthcare workers (12, 13). Studies also show stigma deters the uptake of TB preventive treatment among children with human immunodeficiency virus (HIV) (14) and reduces willingness to be tested and treated for latent TB infection (15).

Despite long-standing anecdotal awareness, stigma was only formally acknowledged in 2017 as a barrier to achieving the World Health Organization (WHO) End TB Strategy targets (16). Since then, multiple high-level commitments—including the United Nations (UN) High Level Meetings on TB in 2018 and 2023, and the Stop TB Partnership Board meetings in 2022 and 2024—have set an explicit goal of eliminating TB stigma by 2027 (17–19).

Traditional stigma-reduction interventions have focused on education, TB support clubs, peer counselling, and health-worker sensitization (20–23), while further innovative approaches such as stigma hackathons are also emerging (19). Evidence from HIV and mental health interventions demonstrates that stigma-reduction initiatives are effective and enhance service uptake and adherence (11). However, the impact of TB-specific stigma-reduction interventions remains poorly quantified and cross-country comparisons challenging due to the limited use of standardised measurement tools (24, 25).

This paper aims to: (a) summarise the implementation feasibility, data quality and key quantitative findings from national TB stigma assessments conducted in 17 high-burden countries during 2020–2025^2^ using established stigma scales collated in a standardised assessment tool developed by the Stop TB Partnership^3^ with Mongolia already published its 2022 assessment results (26), and (b) shaped by community perspectives, discuss the implications of these findings for designing future stigma-reduction interventions and strengthening TB programmes globally.

## 2 Methods

### 2.1 Country assessment framework

To strengthen the effectiveness of TB responses and to support country commitments to eliminate stigma, a standardised TB Stigma Assessment tool was developed in 2019 (27). The tool was developed jointly with KNCV Tuberculosis Foundation, based on a comprehensive review and validation of TB-specific stigma measurable scales (10), in collaboration with TB civil society organisations and affected community networks^4^ (28). The tool focuses on stigma associated with active TB disease (i.e., symptomatic infection) and examines how stigma inhibits access to TB services.

In 2023, the Global Fund formally integrated three indicators from this standardised tool into its grant performance framework—measuring stigma that inhibits service access in three settings: self, healthcare, and community (29).

The assessment process was designed to be civil society-led and nationally endorsed, with guidance and oversight from a multi-stakeholder steering committee comprising the National TB Programme (NTP), lead civil society organisation, technical agencies and representatives of PWTB. This participatory approach aimed to promote the importance of lives experience, building community capacity and shared understanding, validate findings, and co-create action plans for stigma reduction and accountability.

The tool employs both quantitative and qualitative methods to assess the extent and mechanisms through which stigma acts as a barrier to TB services. It also supports countries in developing targeted interventions to ensure services are available, accessible, and acceptable to all.

The tool includes standardised questionnaires based on validated stigma scales, questions measuring service-access-inhibiting stigma, and a standardised EXCEL data-entry file with automated analysis to enable cross-country aggregation. It also includes guidance on the profile and responsibilities of the national stigma expert and the statistician to support the country to conduct the assessment. Together, they supported countries in implementing the assessment according to the standardised guidance. The national stigma expert ensured that translations preserved the intent of the validated stigma scales. The survey statistician designed probabilistic PWTB samples, ensuring the standardised data entry Excel file with automated analysis included in the tool would be used and ensuring data quality including data cleaning and handling of missing data. Finally, all 17 countries received an orientation training from the Stop TB Partnership on how to use the tool and further assessment design, implementation and analytical advice by international experts were also provided to countries upon request.

**Figure 1:**
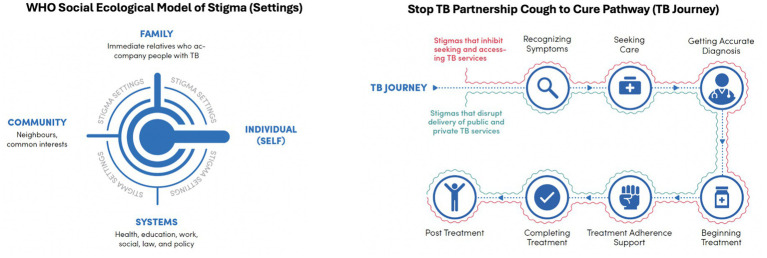
WHO social ecological model of stigma (settings) and Stop TB Partnership cough to cure pathway (TB journey).

The framework explores stigma experienced and observed by PWTB and their families along the TB journey—from recognising symptoms, seeking care, and diagnosis, to treatment initiation, adherence, completion, and post-treatment follow-up. The STP TB journey (Figure 1) was inspired by the TB care cascade (30, 31). Experiences are mapped across four settings—community, family, workplace, and health system—reflecting the social-ecological model (32–34).

Validated stigma scales^5^ were applied to measure stigmatising attitudes among PWTB (self-stigma), family members (secondary stigma), communities, and healthcare workers. Policy makers, legal experts, media representatives, and civil society organisations were also consulted to evaluate (consensus through participatory discussion) the existence, enforcement, and media coverage of laws and policies related to seven TB-relevant rights (structural stigma).^6^ Focus groups and in-depth interviews were conducted to contextualise and triangulate quantitative results.

### 2.2 Data analysis

Assessment reports from 17 countries were reviewed following the structure of the Stop TB Partnership’s standardised tool. Analyses focused on:

1. Feasibility and data quality—assessing whether stigma assessments could be conducted practically and generate robust, nationally representative data.
2. Dimensions of stigma—synthesising quantitative results on:
  - Stigma impact (inhibited access to services across settings);
  - Underlying stigmatising attitudes (as measured by validated scales);
  - Stigma experienced or observed along the TB journey; and
  - Structural stigma (laws, policies, and media coverage).

Data reported in country reports (not raw data of the country) were aggregated to identify common patterns across countries, including:

- Settings where stigma most frequently inhibited service access;
- TB journey stages and population sub-groups (e.g., women, underserved populations, drug resistant TB) most affected; and
- Rights areas requiring urgent policy or legal action.

Standardized questionnaires (validated stigma scales and service-access inhibiting stigma) and standardised data entry with automated analysis used by all 17 countries provided the basis for aggregating country estimates.

Country-level estimates were pooled using sample-size weighting. They were not weighted by national TB burden or general population size. Hence these stigma estimates were descriptive and non-inferential. Sensitivity analyses were not conducted because raw data were unavailable. Countries^7^ that did not provide estimates on a particular indicator were excluded from the aggregated estimates.

Findings were synthesised to inform recommendations for future stigma-reduction interventions and to highlight implications for TB programme design and donor prioritization, particularly in the context of declining global funding and the need for evidence-based, people-centred TB responses.

### 2.3 Patient and public involvement

Across all 17 countries people affected by TB were involved in the design, adaptation, implementation of the stigma assessments at country level. The lead civil society organisations were trained in the theory and operationalisation of the tool and were then involved in the analysis of the national TB stigma assessments and the drafting of this article and are included as authors in this paper.

### 2.4 Limitations

The analysis has several limitations. First, although most country assessments used probabilistic sampling for PWTB, a few did not. These tended to report lower stigma levels. This suggests that pooled stigma levels may be underestimated. Nonetheless, the broad geographical coverage across 17 countries and the pooled PWTB profile—by sex, HIV status, and drug resistance—compares favourably with 2024 global figures.

Second, the samples of family members, community members, and healthcare workers were selected using purposive, non-probabilistic methods (due to resource constraints) and are therefore unlikely to be nationally representative. Hence individual estimates should be interpreted with caution. However, pooling data from 17 countries helps to mitigate this limitation and provides a valuable multi-country perspective.

Third, not all assessments (though most did) engaged PWTB as interviewers. Where healthcare workers conducted interviews, respondents may have underreported stigma in health care settings due to concerns about potential repercussions or being perceived as critical of facilities (self-reporting bias due to perceived social desirability).

Fourth, in some assessments, interviews were conducted by male interviewers, which may have led to underreporting of stigma experiences among women, especially in countries with strong patriarchal social norms (self-reporting bias due to perceived social desirability). This may be because the comfort and openness of women engaging in health studies, particularly relating to sensitive health issues like TB stigma, can be directly impacted by whether the interviewer was male or female, and therefore can contribute to underreporting or non-responsive bias (35–37).

Fifth, findings and recommendations may not apply to PWTB under 18 years of age, as the assessment tool was designed for adults (18 years or above), who account for over 90% of the global TB diagnoses (1). Stigma experienced by children and adolescents warrants further study, as its dynamics—particularly within family settings—may differ substantially and raise additional ethical considerations such as the need for parental consent.

Sixth, findings are descriptive and cross-sectional snapshots and confidence intervals and other inferential statistics were not calculated because raw data were unavailable.

Seventh, despite the questionnaires (the stigma scales in particular) included in the assessment tool (English) were designed to use simple language to both make them easier for interviewees to understand and minimise deviation from the measurement intent of questions when each country adapted them to their own languages and social-cultural context, inconsistencies between countries could still potentially limit the robustness of the pooled findings.

Finally, the tool was not designed to assess stigma related to latent TB infection. Given the likely influence of stigma on preventive therapy uptake and future vaccine introduction, this area requires dedicated investigation.

## 3 Results

### 3.1 Pooled respondent profiles

The 17 high-burden countries included in this review conducted national TB stigma assessments between 2020 and 2025^8^ using the Stop TB Partnership’s standardised tool (standardised questionnaires for stigma scales and attitudes, standardised data entry Excel file with automated analysis). Together, they accounted for 38% (4.05 million) of the estimated 10.7 million global cases of people with TB in 2024 (1). In total, *N* = 26,040 adult (aged 18 years or above)^9^ respondents participated in the quantitative components (mainly face-to-face interviews): *N* = 14,685 PWTB [mean of 17 countries = 864, median = 545, range = 221–3,252], *N* = 4,662 family members [mean = 274, median = 149, range = 26–275], *N* = 4,206 community members [mean = 263, median = 102, range = 20–1,966], and *N* = 2,487 healthcare workers [mean = 146, median = 78, range = 25–640] (Table 1).

**Table 1:** Profile of respondents (pooled data from 17 countries).

	Respondent type						
	People with TB (PWTB)		Family of PWTB		Community/neighbours		Health care workers
*N*	14,685		4,662		4,206		2,487
Region							
Africa	37.5%		59.6%		64.0%		32.6%
Americas	2.5%		0.6%		0.7%		1.2%
Asia	49.9%		37.4%		32.8%		59.1%
Eastern Europe	10.0%		2.3%		2.4%		7.0%
Gender							
Female	38.8%		58.9%		35.7%		63.1%
Male	60.0%		41.0%		58.2%		36.5%
Others	1.3%		0.1%		0.1%		0.1%
Not reported			0.1%		6.0%		0.2%
Age							
18–24	13.0%		6.5%		4.5%		4.9%
25–44	41.6%		39.4%		22.7%		52.4%
45–64	28.7%		20.6%		17.4%		28.9%
65 or over	7.8%		4.4%		2.2%		1.7%
Not reported	8.8%		29.0%		53.2%		12.0%
TB diagnosis		Relationship with PWTB		Know PWTB		Role in health facilities	
Drug-resistant TB	6.4%	Child	19.2%	None	19.3%	Doctor	15.3%
Extra-pulmonary TB	6.9%	Grandchild	0.8%	1	29.5%	Nurse	47.9%
Pulmonary TB	73.6%	Grandparent	1.5%	2 or more	30.4%	Others	31.9%
Not reported	13.1%	Parent	17.4%	Not reported	20.8%	Not reported	4.9%
		Sibling	22.5%				
		Other relatives (including spouse)	31.0%				
Treatment status						Provided care to PWTB	
Completed treatment	38.5%	Not reported	7.5%			Yes	76%
Undergoing treatment	57.0%						
Have not had treatment	1.0%						
Not reported	3.6%						
TB Key and Vulnerable Populations (KVPs) (do not add to 100%, a respondent can belong to none, 2 or more KVPs)							
Rural poor	23.0%						
Urban poor	10.6%						
Indigenous populations	8.4%						
People living with HIV	5.8%						
Former prisoners	4.5%						
People who use drugs	3.5%						
Miners	3.0%						
People with disabilities	2.5%						
Elderly	2.1%						
Refugees/internally displaced persons	1.3%						
Heavy smokers	0.2%						
People with diabetes	0.1%						
People with alcohol dependency	0.1%						
People with mental health issues	0.05%						
Belong to at least 1 KVP *(11 of the 17 countries reported covering 77% of all PWTB respondents)*	66%						

### 3.2 PWTB respondent profile

Half of adult PWTB respondents were from Asia (50%) and over one-third from Africa (38%), with Eastern Europe (10%) and Americas (3%) comprising the remainder. Women accounted for 39%, while respondents living with HIV and those diagnosed with drug-resistant TB represented 5.8 and 6.4%, respectively—figures broadly aligned with global data for adults in 2024 (39%^10^ (1), 5.8%^11^ (1), and 3.6%^12^ (1) respectively). Most assessments employed probabilistic sampling,^13^ typically combining multi-stage cluster sampling of provinces or regions, random selection of treatment centres, and random or convenience sampling from lists of PWTB in those centres.

Among PWTB respondents, 57.0% were undergoing TB treatment and 38.5% completed it. The majority were of working age (25–44 years: 41.6%; 45–65 years: 28.7%). Regarding underserved populations, 23.0% identified as rural poor, 10.6% as urban poor, 8.4% as Indigenous, and smaller proportions as former prisoners (4.5%), people who use drugs (3.5%), miners (3.0%), people with disabilities (2.4%), older adults (2.1%), and refugees or internally displaced persons (1.3%). In 11 countries with detailed underserved populations analyses, two-thirds (66%) of PWTB belonged to at least one underserved population, and in three countries, 20–27% belonged to two or more.

### 3.3 Family respondent profile

Family members were mainly recruited from the same households as PWTB respondents. Guidance in the Stop TB Stigma Assessment tool specified all “adult” (i.e., 18 years or above) members who lived in the same households were eligible to be recruited. Hence, recruitment was guided by two conditions: adult (18 years or above) and lived or living in the same household of the PWTB.^14^ The guidance did not prescribe how those household members meeting these two criteria would be selected and countries did not report on this either. Women constituted 58.9% of participants and 60.1% were aged 25–64. The largest relationship categories were other relatives including spouses^15^ (31.0%) and siblings (22.5%), followed by children (19.2%), parents (17.4%), and grandparents or grandchildren (2.3%).

### 3.4 Community respondent profile

Community members were recruited from neighbourhoods where PWTB lived, mainly through purposive sampling. Women represented 35.7%^16^ (38, 39) of community respondents, and the majority (59.9%) reported knowing at least one PWTB.

### 3.5 Healthcare worker respondent profile

Health care workers (HCW) were largely recruited from facilities in or near communities of PWTB respondents. Women comprised 63.1% of this group, and 81.3% were aged 25–64. Nurses represented nearly half (47.9%) of all respondents, followed by other health professionals (31.9%) and doctors (15.3%). Over three-quarters (76%) reported providing services to PWTB.

Overall, the pooled PWTB dataset appears robust and broadly comparable with global TB data by sex, HIV status, and drug-resistance profile. Because family, community, and health care worker samples were non-probabilistic, their estimates should be interpreted cautiously.

### 3.6 Stigma impact

Across the 17 countries, PWTB respondents reported stigma as barrier to accessing TB services in all five settings assessed. The highest levels were observed for community stigma (25% [median:20%, country range:4–60%]) and self-stigma (23% [median: 16%, range: 6–52%]^17^), followed by stigma in health facilities (18% [median: 15%, range:3–53%]), family (18% [median: 12%, range:3–52%]) and workplace (16% [median: 9%, range:4–49%]). While community was the setting in which stigma had the most inhibiting effects on service access in Africa and Asia, it was self-stigma in Eastern Europe.

Although the tool did not measure overlap between settings, qualitative focus groups indicated that many PWTB experienced stigma in multiple contexts. These findings suggest that stigma acts as a pervasive barrier to TB care—even where diagnosis and treatment are provided free of charge—due to its cumulative and reinforcing effects across settings.

### 3.7 Underlying stigmatising attitudes

A total of 42 items across four stigma scales captured attitudes underlying TB stigma. Each scale was adapted from validated KNCV instruments: self-stigma (PWTB)—“I” statements; secondary stigma (family)—“I” statements; community stigma—“some people” statements (to reduce social desirability bias); and healthcare worker stigma—“some healthcare workers” statement. Four key themes emerged from the pooled data (Table 2).

**Table 2:** Stigma scale items (over 50% shaded, over 66% bolded).

PWTB self-stigma (adapted from Van Rie, 12 items)	% agreed or strongly agreed (*n* = 14,685 PWTB respondents)
**A4—I keep a distance from others to avoid spreading TB germs.**	**75%**
**A9—I choose carefully who I tell about having TB.**	**69%**
A5—I am afraid to tell those outside my family that I have TB.	56%
A1—I feel hurt how others react to knowing I have TB.	56%
A8—I feel guilty because my family has the burden of caring for me.	50%
A3—I feel alone.	45%
A2—I lose friends when I share with them that I have TB.	42%
A10—I feel guilty for getting TB because of my smoking, drinking, or other careless behaviors.	37%
A11—I am worried about having HIV/AIDS.	33%
A7—I am afraid to tell others that I have TB because they may think that I also have HIV/AIDS.	32%
A12—I am afraid to tell my family that I have TB.	29%
A6—I am afraid of going to TB clinics because other people might see me there.	26%

PWTB family secondary stigma (adapted from Arcencio, 10 items)	% agreed or strongly agreed (*n* = 4,662 PWTB family respondents)
A10—I am worried about becoming infected.	52%
A9—I’ve noticed changes in my family member since the TB diagnosis.	50%
A4—My family member hides his/her TB diagnosis from the community.	49%
A3—I hide the fact that my family member has TB from the community.	48%
A1—My family member asks me to keep the TB a secret.	46%
A5—I avoid talking about TB in the presence of other family members or neighbors.	44%
A8—I substitute another word for TB in the conversations with my friends.	39%
A7—I substitute another word for TB in the conversations with my family member.	37%
A2—I feel ashamed because my family member has TB.	29%
A6—I’m afraid that someone will see me at the health care clinic where my relative is being treated.	28%

Community/neighbours stigma against PWTB (adapted from Van Rie, 11 items)	% agreed or strongly agreed (*n* = 4,206 community respondents)
**A5**—**Some people keep their distance from people with TB.**	**69%**
**A1**—**Some people might not want to eat or drink with friends who have TB.**	**69%**
**A2**—**Some people feel uncomfortable being near to those who have TB.**	**67%**
A4—Some people do not want those with TB playing with their children.	66%
A8—Some people are afraid of those with TB.	59%
A9—Some people try not to touch others with TB.	57%
A10—Some people may not want to eat or drink with relatives who have TB.	56%
A3—If a person has TB, some community members will behave differently towards that person for the rest of their life.	50%
A7—Some people do not want to talk to others with TB.	50%
A6—Some people think that those people with TB are disgusting.	46%
A11—Some people prefer not to have those with TB living in their community.	39%

HCW stigma against PWTB (adapted from Corrigan, 9 items)	% agreed or strongly agreed (*n* = 2,487 health care worker respondents)
**A2—Some health care workers feel pity for TB patients.**	**70%**
**A9—Some health care workers think taking TB treatment should be forced if necessary**	**67%**
A7—Some health care workers think it would be best for TB patients to be isolated during the intensive phase of treatment.	61%
A8—Some health care workers feel TB patients are dangerous.	52%
A1—Some health care workers are nervous about treating TB patients.	43%
A4—Some health care workers stay away from TB patients.	39%
A5 - Some health care workers think developing TB is a person’s own fault.	28%
A3—Some health care workers do not like helping TB patients.	27%
A6—Some health care workers feel angry towards TB patients.	25%

*Community stigma:* Stigmatising attitudes were strongest in community samples. Over half of respondents agreed with seven of eleven items, including “do not want to eat and drink with PWTB (69%), “do not let children play with PWTB” (66%), “feel uncomfortable near PWTB” (67%), and “keep a distance from PWTB” (69%).

*Self-stigma:* Among PWTB, four of twelve items were endorsed by more than half. The most common were: “keep a distance from others” (75%), “choose carefully who to tell of TB diagnosis” (69%), “feel hurt by others’ reaction” (56%), and “avoid telling those outside family” (56%).

*Healthcare worker stigma:* Four of nine items were agreed to by over half of healthcare workers, including “feel pity towards PWTB” (70%), “treatment should be forced if necessary” (67%), “best to isolate PWTB during intensive phase” (61%), and “TB patients are dangerous” (52%).

*Family stigma:* Only one of ten items exceeded 50% agreement with families most commonly expressing fear of infection (52%) and tendencies to conceal a relative’s TB diagnosis (49%).

Taken together, these results confirm that stigma remains strongest in community settings and through internalised self-stigma. The endorsement of forced isolation by many healthcare workers is particularly concerning, suggesting possible gaps in infection control training and awareness of PWTB rights. Moreover, one third of PWTB reported fear of being perceived as HIV-positive if their TB status were disclosed—indicating intersectional stigma among PWTB living with HIV.

### 3.8 Stigma experience and observation

Overall, 38% of PWTB reported direct experience of stigma, including non-service-related instances. Reported experiences were lower among family members (19%) and healthcare workers (30%). However, healthcare workers reported the highest rate of observed stigma (24%), exceeding that of PWTB and families (both 20%).

Respondents were asked to identify the settings (community, family, health facility, workplace) and TB journey stages (from symptom recognition to treatment completion) in which stigma occurred. Four consistent patterns emerged (Table 3):

1. *Symptom recognition in the community* was the most frequently reported context for both experienced (9 countries) and observed (12) stigma. Focus group discussions revealed that fear of community stigma led many to ignore persistent symptoms or delay care seeking.
2. *Symptom recognition in health facilities:* ranked second, experienced by PWTB (9) and families (10), reflecting fears of negative treatment by healthcare workers or breach of confidentiality.
3. *Treatment adherence in the community:* was the third most reported, experienced by PWTB (9) and in community (9) suggesting stigma hinders sustained engagement in care.
4. *Stigma among healthcare workers themselves:* was common, with 10 countries reporting experiences and 11 reporting observations of stigma within health settings. This underscores the need to address intra-facility stigma among healthcare workers as part of stigma reduction interventions.

**Table 3:** TB Journey stages and settings where stigma experience and observations were most prevalent (combinations mentioned by more than half of countries shaded, more than two-thirds of countries bolded).

PWTB respondents—top 5 stage-setting combinations stigma experienced and observed by PWTB	Settings (max = 17 countries)							
	Health		Community		Family		Work	
TB journey stages	Stigma experienced	Stigma observed	Stigma experienced	Stigma observed	Stigma experienced	Stigma observed	Stigma experienced	Stigma observed
(1) Recognizing symptoms	9	7	9	8	4	1	1	2
(2) Seeking care	8	6	7	6	0	0	0	0
(3) Getting an accurate diagnosis	7	3	4	3	1	1	0	0
(4) Beginning treatment	6	2	8	6	0	0	1	0
(5) Getting treatment adherence support	2	1	9	7	3	3	1	1
(6) Completing treatment	1	1	0	1	0	0	0	0
(7) Getting post-treatment follow-up services	0	0	0	0	1	0	0	0

PWTB family respondents—top 5 stage-setting combinations stigma experienced and observed by PWTB family	Settings (max = 17 countries)							
	Health		Community		Family		Work	
TB journey stages	Stigma experienced	Stigma observed	Stigma experienced	Stigma observed	Stigma experienced	Stigma observed	Stigma experienced	Stigma observed
(1) Recognizing symptoms	10	3	8	5	3	2	0	0
(2) Seeking care	8	3	7	6	0	2	0	0
(3) Getting an accurate diagnosis	6	4	6	4	1	0	0	0
(4) Beginning treatment	5	5	5	4	1	0	1	1
(5) Getting treatment adherence support	2	0	7	6	1	1	0	1
(6) Completing treatment	1	1	1	3	0	0	0	0
(7) Getting post-treatment follow-up services	0	0	0	0	0	0	0	0

Community respondents—top 5 stage-setting combinations stigma against PWTB observed by community	Settings (max = 17 countries)			
	Health	Community	Family	Work
TB journey stages	Stigma observed	Stigma observed	Stigma observed	Stigma observed
(1) Recognizing symptoms	6	**14**	0	3
(2) Seeking care	3	7	0	1
(3) Getting an accurate diagnosis	4	4	1	0
(4) Beginning treatment	3	7	1	1
(5) Getting treatment adherence support	3	9	0	1
(6) Completing treatment	2	3	0	0
(7) Getting post-treatment follow-up services	1	2	0	0

Health care worker respondents—top setting stigma experienced and observed by health care workers	Settings (max = 17 countries)					
	Hospital (workplace)		Community		Family	
	Stigma experienced	Stigma observed	Stigma experienced	Stigma observed	Stigma experienced	Stigma observed
	10	**11**	4	3	2	2

These findings highlight that stigma exerts the strongest deterrent effects early in the TB journey—during TB symptom recognition—and again during treatment adherence, particularly in community and health facility settings. This pattern aligns with results from the Stop TB Partnership’s *OneImpact* digital platform, which found stigma most pronounced during the testing phase, driven by personal shame, family rejection, and community discrimination (5).

### 3.9 Differences by population sub-groups

Although not required by the standardised tool, most countries analysed results by population sub-groups. Fifteen countries reported gender differences: eleven found women experienced more stigma than men, while four reported the reverse. Where quantified, 22–52% of women and 20–42% of men reported stigma experiences. Qualitative findings indicated women faced greater stigma at home and in communities, while men experienced more in workplaces. For women, stigma often resulted in family abandonment and loss of social and financial support, with wider implications for children’s welfare.

Twelve countries reported higher stigma levels among underserved populations than among the general PWTB population. Those belonging to multiple underserved populations faced compounded stigma. The most frequently affected were PWTB living with HIV (four countries), followed by the rural poor (three countries), people who use drugs (two countries), and migrant workers (2 countries). Stigma against these groups often reflected pre-existing social and economic marginalisation and, in some cases, criminalisation—conditions that limited their ability to seek redress even when legal protection formally existed.

### 3.10 Structural stigma

Most countries expanded their analysis beyond the existence and enforcement of TB-related laws and policies to include how these were portrayed in media. Thirteen countries (Bangladesh, Cambodia, Ghana, Indonesia, Kazakhstan, Malawi, Moldova, Pakistan, Peru, Philippines, Tajikistan, Ukraine, Zimbabwe) assessed seven individual rights most relevant to TB—focusing on law existence, enforcement, and media coverage (Table 4). Three key patterns emerged:

1. Protective frameworks exist in 12 of the 17 countries included in this review (one reported harmful laws and policies and four reported none) but are often non-TB-specific and inconsistently applied across sub-national levels.
2. Enforcement is weak, particularly regarding protection from discrimination, privacy violation, and unsafe workplace conditions.
3. Media coverage of enforcement is weaker still, and in some cases harmful, especially around laws concerning privacy, workplace conditions and involuntary isolation.

**Table 4:** Law, policy and media coverage average scores (0–4): 0 = most harmful to PWTB (existence of harmful laws and policies, enforcement of harmful laws and policies, media coverage of harmful laws and policies at national level), 1 = harmful (sub-national level, not consistent throughout the country), 2 = none, 3 = protective (sub-national level), 4 = most protective of PWTB (national level); (scores lower than 3.0 shaded, scores lower than 2.5 bolded).

TB-related rights	Status of laws and policies (average of countries, n = 13*)					
	Exist		Enforced		Covered by media	
	Laws	Policies	Laws	Policies	Laws	Policies
Rights to Freedom from Discrimination (enacted stigma)	3.5	3.0	2.8	2.6	**2.3**	**2.4**
Rights to access information	3.6	3.4	3.1	3.0	2.8	**2.4**
Rights to access services	3.7	3.6	3.2	3.2	3.1	2.8
Rights to privacy	3.8	3.6	2.9	2.8	**2.1**	**2.3**
Rights to informed consent	3.2	3.6	2.8	3.1	**2.2**	**2.4**
Rights to freedom from arbitrary arrest/detention and involuntary isolation	2.8	3.1	2.8	3.0	**1.8**	**2.1**
Rights to safe workplace	3.1	2.9	**2.3**	**2.4**	**2.1**	**2.2**
Average of 7 rights	3.4	3.3	2.8	2.9	**2.3**	**2.4**

* Bangladesh, Cambodia, Ghana, Indonesia, Kazakhstan, Malawi, Moldova, Pakistan, Peru, Philippines, Tajikistan, Ukraine, Zimbabwe (Azerbaijan, DRC, Mongolia and Nigeria did not report on this).

These structural gaps appear to reinforce health system stigma. The finding that most healthcare workers supported forced isolation likely reflects weak legal enforcement and limited public discussion of the PWTB’s rights. Strengthening both the legal environment and responsible media reporting could thus play critical role in reducing structural stigma.

## 4 Discussion

The key findings from the 17 country assessments included in this review have important implications for both global and national TB responses, particularly for improving the identification of PWTB missed by services and for targeting stigma reduction interventions to ensure equitable access to care.

Stigma was disproportionally borne by women and underserved populations, including people living with HIV, rural communities experiencing poverty, people who use drugs, and migrant workers. These findings are consistent with intersectional stigma frameworks, which highlight how TB stigma compounds existing social and structural vulnerabilities. Although most assessed countries reported constitutional or policy protections applicable to TB, inconsistent enforcement and limited public visibility of these protections undermined their potential to mitigate discrimination and safeguard equitable access to care. Together, these observations suggest that TB stigma operates simultaneously at individual, interpersonal, institutional, and structural levels, requiring responses that are similarly multi-layered.

These findings carry several implications.

First, systematic TB stigma assessments are feasible and provide strategic information to guide programme design. The 17 assessments demonstrate that stigma can be comprehensively measured across TB journey stages, settings, underlying attitudes, laws, policies, media environments, and population sub-groups. Most countries developed stigma reduction action plans based on these findings. Regular assessments, every 3–5 years, led jointly by people affected by TB and NTPs, are recommended to monitor progress and adjust interventions. For example, follow-up assessments in Moldova (2022) and Ukraine (2021) enabled programme adaptations based on measured impact. As more countries undertake such assessments, cross-cutting learning can strengthen evidence-informed practices. The countries should institutionalise the capacity to conduct assessments and, together with people affected by TB, co-create and integrate targeted stigma reduction interventions into TB programming, including through Global Fund Grant Cycle 8. Expansion of current support mechanisms, such as the Challenge Facility for Civil Society and the OneImpact Community-led Monitoring, will be critical. Finally, TB stigma indicators should be incorporated into global monitoring frameworks, including WHO’s Global TB Report, using standardised data collection tools. The TB Stigma Portal (Stop TB Partnership, 2024) could further support harmonised monitoring and knowledge exchange.

Second, the design, implementation, and budgeting of TB stigma interventions must shift from generic awareness-raising approaches toward more focused, context-responsive strategies explicitly aligned with observed drivers of stigma. Programming should prioritise interventions at key TB journey stages, symptom recognition, testing, and treatment adherence, and directly address self-isolation, community avoidance, and coercive attitudes toward isolation. Dedicated budgets are essential. Interventions should respond to differential stigma experiences among men, women, and underserved populations, as well as to stigma expressed within health settings. Targeting should reflect specific underlying attitudes, service delivery, environments (self, household, community, health facilities), and the broader legal and media context shaping public perceptions.

Third, legislation and policy gaps must be addressed as part of stigma reduction efforts. In settings lacking protective legal frameworks, interventions should include advocacy for workplace protection, anti-discrimination safeguards, particularly regarding safe workplaces and job security for PWTB. In countries where protective frameworks exist—whether TB-specific or embedded in broader human rights legislation—interventions should focus on strengthening and increasing awareness and media visibility of rights related to non-discrimination, privacy, and protection from involuntary isolation. Without enforcement and public awareness, legal protections alone are insufficient to counter stigma.

Fourth, stigma reduction efforts must expand to include latent TB infection. The demonstrated impact of stigma on accessing TB disease services suggests similar barriers for latent TB testing and preventive treatment, particularly as shorter preventive regimens and new vaccines become available. Given that asymptomatic individuals may face disease-associated stigma despite not having active TB, assessment tools and interventions should be adapted to explore and address these emerging dynamics.

Fifth, future assessments should include sufficiently powered, disaggregated analyses by population subgroup. Women, men, underserved populations, and where feasible, children should be represented with adequate sample sizes (e.g., at least 40 respondents per priority subgroup) to enable robust analysis. Recommendations and interventions must respond not only to the general PWTB population but also to groups experiencing distinct or intensified forms of stigma. The standardised assessment tool could be further strengthened to provide clearer methodological guidance on subgroup-specific analysis and intervention design.

Sixth, additional research is warranted, including meta-analysis synthesising quantitative stigma scale data across settings (self, family, community, HCW stigma), and across TB journey stages. Further work could also address methodological and contextual limitations identified in this study.

In summary, these findings demonstrate that TB stigma is not peripheral but central to the care cascade. Targeted, evidence-based stigma reduction interventions—integrated into TB programming, supported by enforceable legal protection, and informed by systematic assessment—are essential to improving TB case finding, strengthening equitable access to care, saving lives, and ultimately, ending TB-related stigma globally.

## 5 Conclusion

Ending TB stigma is both a moral imperative and a strategic necessity for achieving global TB targets. This review demonstrates that the Stop TB Partnership standardised stigma assessment tool is feasible, practical, and capable of generating robust, policy-relevant evidence when implemented through civil society-led partnerships with National TB Programmes.

Findings show that stigma remains a pervasive barrier across multiple settings and stages of the TB journey, particularly during symptom recognition and treatment adherence. Interventions must therefore be targeted, data driven, and tailored to specific populations, settings, and underlying attitudes. Legal and policy protections must not only exist but be enforced and publicly visible.

Future efforts should prioritise disaggregate analysis by population sub-groups, expand measurement to latent TB infection, and institutionalise stigma monitoring within national and global reporting frameworks. Sustained domestic and external investment, including through mechanisms such as the Global Fund and the Stop TB Partnership Challenge Facility for Civil Society, is essential to embed stigma reduction within TB programming and accelerate progress toward ending TB and TB-related stigma.

## Data Availability

The original contributions presented in the study are included in the article/supplementary material, further inquiries can be directed to the corresponding author.

## Footnotes

1 Word Health Organisation’s estimated 10.7 million global TB incidence in 2024 and countries collectively reported 8.3 million cases of new diagnosis in the same year, suggesting 22% of TB cases were missed in 2024 globally (1).

2 17 national level assessments were conducted between 2020 and 2025: Africa (5) - Democratic Republic of Congo (2023), Ghana (2020), Malawi (2024), Nigeria (2023), Zimbabwe (2024); Asia (8) - Bangladesh (2024), Cambodia (2025), Indonesia (2022), Kazakhstan (2025), Mongolia (2022), Pakistan (2024), Philippines (2025), Tajikistan (2025); Eastern Europe (3) - Azerbaijan (2024), Moldova (2025), Ukraine (2024) [Moldova conducted the first assessment in 2022 and Ukraine in 2021]; Americas (1) - Peru (2024).

3 Stigma assessment is a population-based tool to measure periodically level of stigma before and after interventions and differs from OneImpact, which is a community-led monitoring tool that enables people affected by TB to report barriers to accessing TB services, including stigma, in real time.

4 Civil society organisations: Activists’ Coalition on TB Asia-Pacific (ACT! Asia-Pacific), African Coalition on TB (ACT), Civil Society Movement Against Tuberculosis – Sierra Leone (CISMAT-SL), Dynamique de la Réponse d’Afrique Francophone sur la Tuberculose (DRAF TB), Eastern Africa National Networks of AIDS and Health Service Organizations (EANNASO), International Council of Nurses, KELIN, Frontline AIDS, Global Coalition of TB Activists (GCTA), Global Fund to Fight AIDS, Tuberculosis and Malaria, National TB Programme representatives, REACH, TB Europe Coalition (TBEC), TBpeople, KNCV, The Work for Change, UNAIDS, University of Sydney, Woolcock Institute of Medical Research, USAID and WHO.

5 Stigma scales: (1) PWTB self-stigma (12 items) – adapted from Van Rie TB Patient Scale; (2) PWTB family secondary stigma (10 items) – adapted from Arcencio TB Stigma Scale; (3) Community stigma against PWTB (11 items) – adapted from Van Rie TB Community Stigma Scale; (4) Health care worker stigma against PWTB (9 items) – adapted from Corrigan TB Health Care Worker Stigma Scale; KNCV (10).

6 7 TB-related rights include: (1) Rights to Freedom from Discrimination (enacted stigma), (2) Rights to Access Information, (3) Rights to Access Services, (4) Rights to Privacy, (5) Rights to Informed Consent, (6) Rights to Freedom from Arbitrary Arrest/Detention and Involuntary Isolation, (7) Rights to Safe Workplace. This differs from legal environment assessments (see Stop TB Partnership’s Legal Environment and Human Rights Scorecard, https://www.stoptb.org/sites/default/files/imported/page/file/17199/file_17199.pdf) and focuses on not just existence and enforcement of laws and policies but media coverage which is often how stigma spreads and is reinforced (structural stigma).

7 All countries provided estimates on the 5 service-access inhibiting stigma level and all items of the 4 stigma scales, except Nigeria (self-stigma inhibiting services access) and Mongolia (community and health care worker stigma scales).

8 Funded by the country’s Global Fund country grants or the Challenge Facility for Civil Society programme of the Stop TB Partnership. Global Fund grant implementers (Principal Recipients and Sub-Recipients) were selected through a competitive and transparent country process overseen by the Country Coordination Mechanisms and the Global Fund Country Teams. Stop TB Partnership Challenge Facility for Civil Society grantees were selected through an open call and independent review panel evaluation. All countries secured approvals by their country ethics review boards. Countries conducted their assessment in different times during the 2020–2025 period and most countries completed their assessments in 9–12 months.

9 Inclusion criteria recommended in the Stop TB Partnership TB stigma assessment guidance - aged 18 or above (PWTB diagnosed with TB before, family members lived in the same houses as the PWTB interviewed, leaders and neighbours in the same community where interviewed PWTB lived, health care workers in health facilities near where the interviewed PWTB lived).

10 2024 global total estimated TB incidence 10.7 million (36% adult women, 56% adult men and 8% children). Hence 39% [36% / (36% + 56%) x 100%] of adults were women.

11 Global total of 619,000 estimated incidence of TB among PLHIV in 2024.

12 Global total of 390,000 estimated incidence of rifampicin-resistant TB (RR-TB) and multidrug-resistant TB (MDR-TB, defined as resistance to both rifampicin and isoniazid) in 2024.

13 While 12 of the 17 countries reported probabilistic random sampling methods were used for their PWTB samples, three countries reported non-probabilistic sampling methods were used and two did not provide enough sampling methods information in their assessment reports. Countries did not include response rates in their assessment reports.

14 Following the Stop TB Partnership tool guidance (1 family member per household where the interviewed PWTB resided) all 17 countries reported family respondents were mainly recruited from the same households of the PWTB respondents. However, none provided in their reports detailed breakdowns on proportion of the family respondents from outside the households.

15 Spouse was not included in the response category in the Stop TB Partnership assessment tool questionnaire (last question in the family questionnaire—relationship with the PWTB) as a result of concerns raised in pilots during the tool development phase that could be too sensitive and might make respondents in countries with strong gender norms less likely to reveal the true stigma experience. However, focus groups of the 17 assessments included in this review not only did not find such bias and these countries subsequently informed the authors of this paper most of the “other relatives” category were in fact spouses. The Stop TB Partnership will update its tool to include spouse as a separate response category.

16 Countries from Africa had lower % of female community respondents than other regions, potentially related to social norms around autonomy, availability and willingness to participate in that region. This is consistent with research findings relating to clinical trials, authorship, reporting and health service use particularly on contexts of sensitive health issues like TB stigma.

17 Based on 11,433 respondents (16 countries, Nigeria did not report on this indicator).

## Author’s note

Rhea Lobo, Tanaka Mukuhwa, Maxime Lunga, and Bertille Kayowa are TB survivors.

## References

[1] World Health Organisation *Global Tuberculosis Report 2025* (2025) Available online at: https://iris.who.int/server/api/core/bitstreams/e97dd6f4-b567-4396-8680-717bac6869a9/content (Accessed November 19, 2025)

[2] Craig GM Daftary A Engel N O’Driscoll S Ioannaki A. Tuberculosis stigma as a social determinant of health: a systematic mapping review of research in low incidence countries. Int J Infect Dis. (2016) 56:90–100. doi: 10.1016/j.ijid.2016.10.011 27810521

[3] Daftary A Mitchell EMH Reid MJA Fekadu E Goosby E. To end TB, first-ever high-level meeting on tuberculosis must address stigma. Am J Trop Med Hyg. (2018) 99:1114–6. doi: 10.4269/ajtmh.18-0591, 30226149 PMC6221214

[4] Citro B Soltan V Malar J. Building the evidence for a rights-based, people-centered, gender-transformative tuberculosis response: an analysis of the stop TB partnership community, rights, and gender tuberculosis assessment. Health Hum Rights. (2021) 23:253–67. 34966240 PMC8694305

[5] Stop TB Partnership *OneImpact: for a TB Free Community, Global Impact Report* (2025) Available online at: https://www.stoptbpartnershiponeimpact.org/assets/images/newimages/ResourceLibrary/resourcelibrarypdf/Global%20Report%20Final.pdf (Accessed June 24, 2025)

[6] Goffman E. Stigma: Notes on the Management of Spoiled Identity. Englewood Cliffs: Prentrice-Hall (1963).

[7] Weiss MG Ramakrishna J Somma E. Health-related stigma: rethinking concepts and interventions. Psychol Health Med. (2006) 11:277–87. doi: 10.1080/13548500600595053, 17130065

[8] Hatzenbuehler ML Phelan JC Link BG. Stigma as a fundamental cause of population health inequalities. Am J Public Health. (2013) 103:813–21. doi: 10.2105/AJPH.2012.301069, 23488505 PMC3682466

[9] Macintyre K Bakker MI Bergson S Bhavaraju R Bond V Chikovore J . Defining the research agenda to measure and reduce tuberculosis stigmas. Int J Tuberculosis Lung Dis. (2017) 21:87–96. doi: 10.5588/ijtld.17.0151, 29025490

[10] KNCV TB Stigma Measurement Guidance (2018). Available online at: https://www.challengetb.org/publications/tools/ua/TB_Stigma_Measurement_Guidance.pdf (Accessed August 02, 2018)

[11] Majeed T Hopkin G Wang K Nepal S Votruba N Gronholm P . Anti-stigma interventions in low-income and middle-income countries: a systematic review. eClinMed. (2024) 72:102612. doi: 10.1016/j.eclinm.2024.102612, 38707913 PMC11066569

[12] Fenta MD Ogundijo OA Warsame AAA Belay AG. Facilitators and barriers to tuberculosis active case findings in low- and middle-income countries: a systematic review of qualitative research. BMC Infect Dis. (2023) 23:515. doi: 10.1186/s12879-023-08502-7, 37550614 PMC10405492

[13] Bodur MS Çil B. A research on healthcare professionals' stigma towards tuberculosis patients. Thoracjc Res Pract. (2025) 26:88–96. doi: 10.4274/ThoracResPract.2024.24079, 39930730 PMC12047200

[14] Amuge PM Ndekezi D Mugerwa M Bbuye D Rutebarika DA Kizza L . Facilitators and barriers to initiating and completing tuberculosis preventive treatment among children and adolescents living with HIV in Uganda: a qualitative study of adolescents, caretakers and health workers. AIDS Res Ther. (2024) 21:59. doi: 10.1186/s12981-024-00643-2, 39210349 PMC11363537

[15] Kılıç A Zhou X Moon Z Hamada Y Duong T Layton C . A systematic review exploring the role of tuberculosis stigma on test and treatment uptake for tuberculosis infection. BMC Public Health. (2025) 25:628. doi: 10.1186/s12889-024-20868-0, 39953433 PMC11829483

[16] World Health Organisation *Ethics Guidance for End TB Strategy Implementation* (2017) Available online at: https://iris.who.int/bitstream/handle/10665/254820/9789241512114-eng.pdf?sequence=1 (Accessed January 17, 2025)

[17] UN General Assembly, UN High Level Meeting on Tuberculosis *Political Declaration of the High-Level Meeting of the General Assembly on the Fight Against Tuberculosis* (2023) Available online at: https://www.stoptb.org/sites/default/files/imported/wysiwyg_block/webadmin/cms/docs/Political-Declaraion-on-the-Fight-against-Tuberculosis.pdf (Accessed April 19, 2024)

[18] Stop TB Partnership *Global Plan to End TB* (2022) Available online at: https://www.stoptb.org/sites/default/files/imported/document/global_plan_to_end_tb_2023-2030.pdf (Accessed April 19, 2024)

[19] Stop TB Partnership *38th Board Meeting Decision Points, Decision 38–2* (2024) Available online at: https://www.stoptb.org/sites/default/files/documents/38%20Stop%20TB%20BOARD%20MEETING.pdf (Accessed March 29, 2025)

[20] Thornicroft G Mehta N Clement S Evans-Lacko S Doherty M Rose D . Evidence for effective interventions to reduce mental health-related stigma and discrimination. Lancet. (2016) 387:1123–32. doi: 10.1016/S0140-6736(15)00298-6, 26410341

[21] Foster I Galloway M Human W Anthony M Myburgh H Vanqa N . Analysing interventions designed to reduce tuberculosis-related stigma: a scoping review. PLoS Global Public Health. (2022) 2:e0000989. doi: 10.1371/journal.pgph.0000989, 36962638 PMC10022226

[22] Nuttall C Fuady A Nuttall H Dixit K Mansyur M Wingfield T. Interventions pathways to reduce tuberculosis-related stigma: a literature review and conceptual framework. Infect Dis Poverty. (2022) 11:101. doi: 10.1186/s40249-022-01021-8, 36138434 PMC9502609

[23] Macdonald SFH France NF Hodgson I Ali F. Piloting “from the inside out” — a toolkit addressing tuberculosis-related self-stigma. BMC Glob Public Health. (2024) 2:31. doi: 10.1186/s44263-024-00062-5 39681934 PMC11622890

[24] Sommerland N Wouters E Mitchell EMH Ngicho M Redwood L Masquillier C . Evidence-based interventions to reduce tuberculosis stigma—a systematic review. Int J Tuberc Lung Dis. (2017) 21:81–6. doi: 10.5588/ijtld.16.0788, 29025489

[25] Bergman AJ McNabb K Farley JE. A systematic review and psychometric appraisal of instruments measuring tuberculosis stigma in sub-Saharan Africa. Stigma Health. (2024) 9:81–93. doi: 10.1037/sah0000328, 38420140 PMC10901500

[26] Tsogt B Denholm JT Dambaa N Sambuu T Tsegeen N Munkhjargal G . TB-related stigma is widely prevalent among people with TB and carers in Mongolia. IJTLD Open. (2025) 2:420–6. doi: 10.5588/ijtldopen.25.0174, 40657263 PMC12248404

[27] Stop TB Partnership *TB Stigma Assessment Tool* (2019) Available online at: https://www.stoptb.org/tb-stigma-assessment-tool (Accessed November 8, 2019)

[28] Stop TB Partnership *TB Stigma Assessment Implementation Handbook* (2019) Available online at: https://www.stoptb.org/tb-stigma-assessment-tool (Accessed November 8, 2019)

[29] Global Fund *Technical Brief: Removing Human Rights-Related Barriers to TB Services - Allocation Period 2023-2025* (2023) Available online at: https://resources.theglobalfund.org/media/14341/cr_removing-barriers-to-tb-services_technical-briefing-note_en.pdf (Accessed April 19, 2024)

[30] Subbaraman R Nathavitharana RR Mayer KH Satyanarayana S Chadha VK Arinaminpathy N . Constructing care cascades for active tuberculosis: a strategy for program monitoring and identifying gaps in quality of care. PLoS Med. (2019) 16:e1002754. doi: 10.1371/journal.pmed.1002754, 30811385 PMC6392267

[31] Vesga JF Hallett TB Reid MJA. Assessing tuberculosis control priorities in high-burden settings: a modelling approach. Lancet Global Health. (2019) 7:e585–95. doi: 10.1016/S2214-109X(19)30037-3, 30904521

[32] Bronfenbrenner U. Toward an experimental ecology of human development. Am Psychol. (1977) 32:513–53. doi: 10.1037/0003-066x.32.7.513

[33] McLeroy KR Bibeau D Steckler A Glanz K. An ecological perspective on health promotion programmes. Health Educ Q. (1988) 15:351–77. doi: 10.1177/109019818801500401 3068205

[34] World Health Organisation *Violence prevention unit: approach, objectives, and activities, 2022-2026* (2021) Available online at: https://cdn.who.int/media/docs/default-source/documents/social-determinants-of-health/who_2022_plv_strategy_2022-2026_finalfile.pdf?sfvrsn=c819ff54_3 (Accessed January 17, 2025)

[35] Davis RE Couper MP Janz NK Caldwell CH Resnicow K. Interviewer effects in public health surveys. Health Educ Res. (2010) 25:14–26. doi: 10.1093/her/cyp046, 19762354 PMC2805402

[36] Haber N. Robyn P. J. Hamadou S. Yama G. Hien H. Louvouezo D. . (2018). Surveyor gender modifies average survey responses in four sub-Saharan African countries. arXiv [Preprint] Doi: 10.48550/arXiv.1810.01981

[37] Vollmer N Singh M Harshe N Valadez JJ. Does interviewer gender influence a mother's response to household surveys about maternal and child health in traditional settings? A qualitative study in Bihar, India. PLoS One. (2021) 16:e0252120. doi: 10.1371/journal.pone.0252120, 34133433 PMC8208568

[38] Zeleke GA Mekonen EG Terefe B . Barriers to healthcare access among women in sub-Saharan Africa: a pooled analysis of multi-country demographic and health survey (2019–2023) data. PLoS One. (2026) 21:e0331328. doi: 10.1371/journal.pone.0331328 41712586 PMC12919819

[39] Aboagye RG Essuman MA Salihu T Seidu AA Hagan JE Baiden F . Association between the survey-based women's empowerment (SWPER) index and barriers to healthcare in sub-Saharan Africa. Int Health. (2025) 17:734–44. doi: 10.1093/inthealth/ihaf023, 40390700 PMC12406787

